# Transcriptomic and Functional Characterization of *ClHsf8* Reveals Key Mechanisms of Heat Stress Response in *Cunninghamia lanceolata*

**DOI:** 10.3390/plants15081150

**Published:** 2026-04-09

**Authors:** Yuan Ji, Liming Zhu, Yuming Luo, Xueyan Zheng, Weihuang Wu, Jisen Shi, Renhua Zheng, Jinhui Chen

**Affiliations:** 1 Jiangsu Collaborative Innovation Center of Regional Modern Agriculture & Environmental Protection, Huaiyin Normal University, Huai’an 223300, China; jsjiyuan@163.com (Y.J.);; 2 Key Laboratory of Forest Genetics & Biotechnology of Ministry of Education, CoInnovation Center for Sustainable Forestry in Southern China, Nanjing Forestry University, Nanjing 210037, China; 3 National Germplasm Bank of Chinese Fir at Fujian Yangkou Forest Farm, Nanping 353211, China; 4 State Key Laboratory of Tree Genetics and Breeding, Research Institute of Tropical Forestry, Chinese Academy of Forestry, Guangzhou 510520, China; 5 Fujian Academy of Forestry, Fuzhou 350012, China

**Keywords:** *Cunninghamia lanceolata*, heat stress, *ClHsf8*, abiotic stress tolerance

## Abstract

*Cunninghamia lanceolata* (*C. lanceolata*), a pivotal economic timber species in southern China, faces increasing threats from global warming and heat stress. Due to limited knowledge regarding its stress response mechanisms, uncovering the molecular basis of heat tolerance is crucial for breeding resilient varieties. Therefore, the objective of this study was to elucidate the physiological and molecular mechanisms of *C. lanceolata* in response to heat stress. In this study, we performed a time-series transcriptomic analysis on leaves of *C. lanceolata* ‘6421’ seedlings exposed to heat stress (39 °C) for 0, 1, 4, 8, 12, and 16 h. A total of 1130 differentially expressed genes (DEGs) were identified, with functions primarily enriched in signal transduction, protein folding, and the MAPK and NF-kappa B signaling pathways. Weighted gene co-expression network analysis (WGCNA) revealed a complex regulatory network, identifying *ClHsf8* as a central hub transcription factor. To validate its function, *ClHsf8* was cloned and overexpressed in tobacco (*Nicotiana benthamiana*). Under heat stress conditions, transgenic plants exhibited enhanced thermotolerance compared to wild-type controls, characterized by significantly higher activities of antioxidant enzymes (SOD, POD, and CAT) and reduced accumulation of MDA and H_2_O_2_. Our findings elucidate the molecular regulatory mechanisms of *C. lanceolata* in response to high temperatures and demonstrate the functional role of *ClHsf8* in conferring heat tolerance, providing a theoretical foundation for the genetic improvement of heat-resilient cultivars.

## 1. Introduction

*Cunninghamia lanceolata* (*C. lanceolata*) is a major economic timber species widely distributed across approximately ten provinces in southern China [1]. It is extensively cultivated due to its fast growth and high-quality wood, playing a vital role in ecological construction and timber production. As one of the most important coniferous tree species in China, *C. lanceolata* faces significant challenges from rising global temperatures and frequent extreme heat events [2]. Heat stress has become a critical environmental factor limiting its growth and reproduction, seriously threatening stable production and sustainable utilization of *C. lanceolata* resources [3].

Plants’ adaptation to heat stress largely depends on complex molecular regulatory networks, among which heat shock transcription factors (*Hsfs*) play a central role in sensing and responding to high-temperature stimuli [4]. *Hsfs* regulate the expression of heat shock proteins (Hsps) and other defense-related genes, protecting cellular protein structures and maintaining homeostasis, thereby enhancing plant thermotolerance. Previous studies have demonstrated that the *Hsf* gene family is widespread in various plants such as *A. thaliana* and *P. bournei*, and is involved in abiotic stress responses [5,6]. However, systematic identification and functional characterization of the *Hsf* gene family in *C. lanceolata* remain limited.

However, the molecular mechanisms underlying heat tolerance in *C. lanceolata* remain largely unexplored. Therefore, the objective of this study was to elucidate the physiological and molecular mechanisms of *C. lanceolata* in response to heat stress. To gain a deeper understanding of the molecular response mechanisms of *C. lanceolata* to heat stress, this study evaluated the physiological changes in the ‘6421’ cultivar under both 39 °C and 42 °C, and subsequently performed transcriptome sequencing to analyze gene expression dynamics specifically at 39 °C. Key differentially expressed genes were identified through bioinformatics analyses, highlighting *ClHsf8* as a crucial regulator in the heat stress response. Furthermore, transgenic tobacco plants overexpressing *ClHsf8* were generated to validate its role in enhancing heat tolerance. This study not only elucidates the molecular regulatory mechanisms of heat tolerance in *C. lanceolata* but also provides a theoretical foundation and genetic resources for molecular breeding of heat-resistant varieties.

## 2. Results

### 2.1. Phenotypic and Stomatal Changes Under Heat Stress

**Figure 1:**
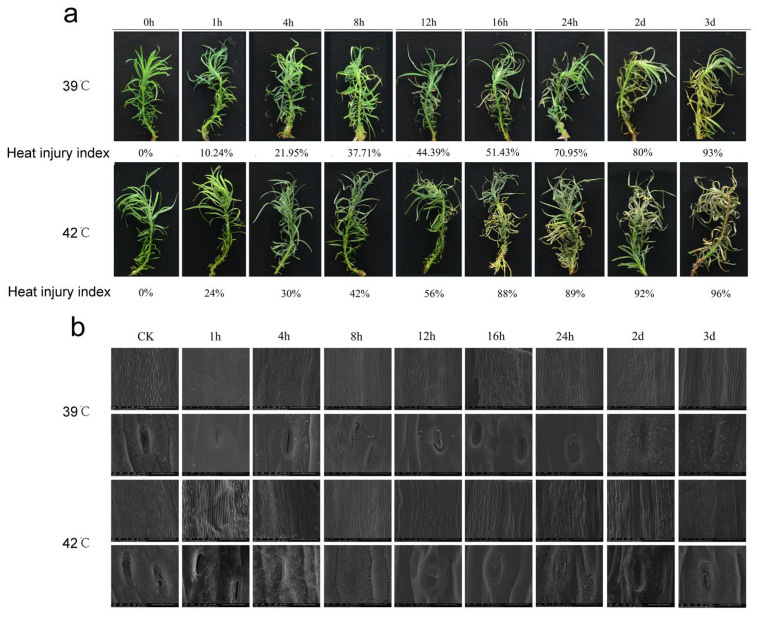
Phenotypic changes and stomatal morphology of *C. lanceolata* under heat stress. (**a**) Phenotypic changes and heat damage index under 39 °C and 42 °C treatments; (**b**) Scanning electron microscopy of leaf stomata under 39 °C and 42 °C treatments.

Under 39 °C and 42 °C heat stress, phenotypic changes in *C*. *lanceolata* tissue-cultured seedlings were monitored from 0 h to 3 d. As shown in Figure 1a, the heat injury index increased with treatment duration, reflecting cumulative physiological damage and loss of cellular homeostasis. At 39 °C, seedlings maintained relative integrity during the early stages, with leaves beginning to yellow at 16 h (Heat Injury Index: 51.43%) and wilting significantly by 24 h (70.95%). By day 3, the plants showed severe desiccation with a mortality rate of 93%. In contrast, the 42 °C treatment induced much more rapid deterioration. Yellowing was evident as early as 12 h (56%), and the heat injury index spiked sharply to 88% by 16 h—a level of damage that took nearly two days to reach under 39 °C. This accelerated progression confirms that 42 °C exceeds the seedling’s physiological tolerance threshold much faster than 39 °C, leading to rapid tissue necrosis.

To investigate the physical basis of this damage, scanning electron microscopy was used to observe stomatal behavior (Figure 1b). The stomata exhibited a dynamic pattern of initial opening followed by closure, which was directly correlated with the melting of epicuticular wax. At 39 °C, stomata expanded within 1 h and peaked at 4 h. However, as stress persisted, the wax layer began to dissolve, forming filamentous networks by 8 h and completely sealing the stomata by 16 h. At 42 °C, this structural collapse occurred significantly earlier; although stomata opened at 1 h, significant wax melting was visible by 4 h, and the stomata were fully sealed by 8 h. The melting of epicuticular wax represents irreversible structural damage. While the subsequent sealing of stomata may limit water loss, it physically blocks gas exchange, likely exacerbating metabolic disruption and accelerating the physiological decline observed in the phenotypic analysis.

### 2.2. Effects of Heat Stress on the Physiology of C. lanceolata

The total Chlorophyll content of *C. lanceolata* seedlings gradually decreased with increasing duration of high temperature stress (Figure S1a). No significant differences were observed within the first 12 h, but a marked decline occurred after 16 h, especially under 42 °C. Under 39 °C, a significant decrease was evident at 24 h.

Malondialdehyde (MDA) content increased rapidly during the initial 0–4 h, reaching a peak at 4 h, followed by a gradual decline (Figure S1b). Throughout the treatment, MDA levels were consistently lower under 42 °C than under 39 °C, suggesting less membrane lipid peroxidation at the higher temperature. Superoxide dismutas (SOD) activity remained relatively stable during early treatment at 39 °C, increased from 4 to 12 h, and then plateaued; under 42 °C, it first increased and then decreased with prolonged exposure (Figure S1c), indicating possible enzyme inhibition at higher temperatures. Proline content showed a continuous increase over time, with significantly higher levels under 42 °C than 39 °C (Figure S1d).

Collectively, *C. lanceolata* responded to heat stress with chlorophyll degradation, increased oxidative stress, modulation of antioxidant enzyme activity, and accumulation of osmolytes such as proline. These physiological responses were significantly more pronounced at 42 °C, indicating that 42 °C represents a severe stress intensity that may exceed the physiological tolerance limit of the seedlings over prolonged exposure.

### 2.3. Transcriptomic Sequencing and Correlation Analysis

The physiological analysis indicated that while 42 °C triggered a more intense response, it also led to rapid damage that could obscure specific regulatory mechanisms. Therefore, 39 °C was selected as the optimal temperature to capture dynamic transcriptional regulation without inducing immediate lethality. To investigate the transcriptomic response under this condition, RNA sequencing was performed on leaf samples collected at six time points (0, 1, 4, 8, 12, and 16 h), generating a total of 142 Gb raw data and 134 Gb clean data after filtering. The sequencing depth per sample ranged from 6.1 to 9.8 Gb (Table S1). Transcript assembly yielded 21,331 predicted transcripts, mostly ranging from 500 to 2000 bp in length. Pearson correlation analysis confirmed high consistency among replicates (Figure S2a), grouping the samples into three highly correlated modules (R^2^ > 0.7): control (CK), short-term stress (1–4 h), and long-term stress (8–16 h). The differences in correlation between modules reflect dynamic gene expression changes induced by heat stress. Principal component analysis (PCA) further revealed clear separation among the three groups (Figure S2b), with control, short-term, and long-term stress samples forming distinct clusters. These results support the Pearson correlation analysis and indicate significant time-dependent transcriptomic responses to heat stress.

### 2.4. Gene Expression Dynamics and Functional Enrichment Under Heat Stress

**Figure 2:**
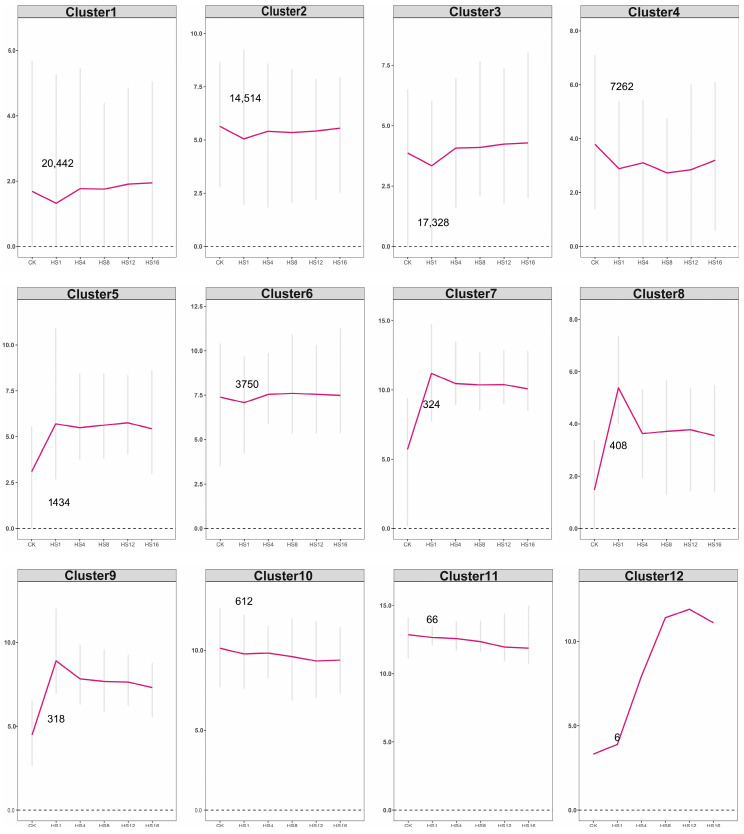
Cluster diagram of gene expression pattern under high temperature stress.

To investigate gene expression dynamics under heat stress, 11,094 differentially expressed genes (DEGs) were clustered based on TPM values using K-means, resulting in 12 distinct expression profiles (Figure 2). These profiles were grouped into three main patterns: up-and-down (clusters 5, 7, 8, 9, 12), down-and-up (clusters 1, 2, 3, 4, 6), and gradual decline (clusters 10, 11). Notable trends include early peaks at 1 h in clusters 5, 7, 8, and 9, a peak at 12 h in cluster 12, and transient downregulation at 1 h in clusters 1, 2, 3, and 6 followed by recovery.

**Figure 3:**
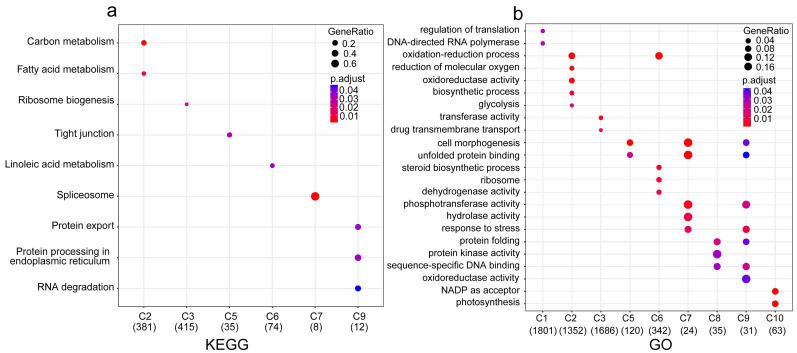
KEGG and GO enrichment analysis of the identified gene clusters under high-temperature stress. (**a**) KEGG pathway enrichment analysis of specific gene clusters. (**b**) GO enrichment analysis of specific gene clusters. The *x*-axis represents the different gene clusters (C1–C10), with the numbers in parentheses indicating the total number of genes within each cluster. The *y*-axis represents the significantly enriched KEGG pathways or GO terms.

Functional enrichment analyses further elucidated the roles of these clusters. GO enrichment identified nine functionally distinct groups (Figure 3b), including translational regulation (cluster 1), redox processes and steroid biosynthesis (clusters 2 and 6), glycosyltransferase activity and transport (cluster 3), cell morphogenesis and unfolded protein binding (clusters 5 and 9), stress response and protein folding (clusters 7 and 8), and photosynthesis-related functions (cluster 10). Clusters 7–9, enriched in stress sensing, transcriptional regulation, and cellular maintenance, are especially pertinent to *C. lanceolata*’s heat stress response.

KEGG pathway analysis revealed significant enrichment in six clusters (Figure 3a). Notably, pathways related to ribosome biogenesis and linoleic acid metabolism were enriched in clusters 3 and 6, which exhibited decreased expression patterns. This down-regulation is consistent with a rapid, transient decrease in general cellular resources in order to activate adaptation to heat stress. Conversely, pathways such as tight junctions (cluster 5), spliceosome function (cluster 7), and protein export with ER protein processing and RNA degradation (cluster 9) were also significantly enriched. Together with GO results, these findings suggest that *C. lanceolata* adapts to heat stress through a strategic reallocation of cellular resources: transiently suppressing normal growth processes to prioritize the induction of heat-responsive protein synthesis (e.g., heat shock proteins) and the maintenance of proteostasis.

### 2.5. Weighted Gene Co-Expression Network Analysis

**Figure 4:**
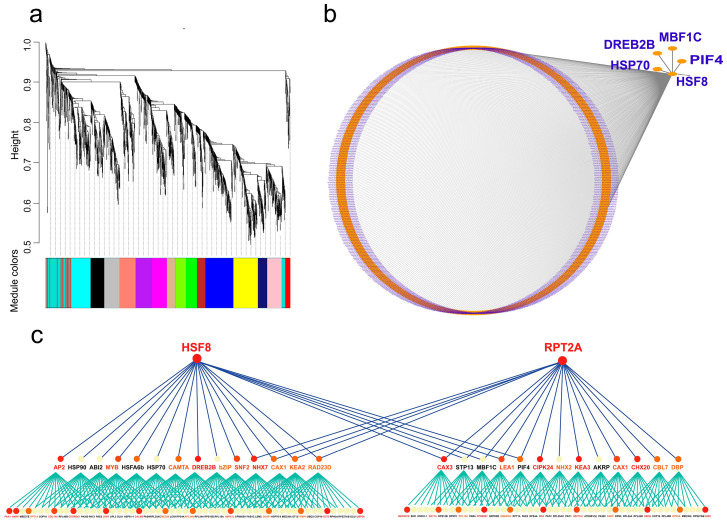
Multiscale gene co-expression and regulatory network architecture of Clion under high temperature stress: (**a**) Gene co-expression clustering module diagram. (**b**) Regulatory network analysis of gene sets of distinctive modules. (**c**) Multilevel transcriptional regulatory network integrating transcription factors and their downstream target genes.

To unravel the regulatory network of heat-responsive genes, WGCNA was performed using TPM values of 1130 core DEGs. A co-expression network was constructed with a soft threshold of 12 and correlation coefficient R^2^ > 0.8, yielding 16 distinct modules visualized as a clustering tree, each colored uniquely to represent gene co-expression clusters. Module-trait correlation analysis was conducted to explore the regulatory network of DEGs under heat stress and identify key response time points. A heatmap of 16 modules showed distinct correlations with different time points: the Turquoise module was most strongly correlated with the control (CK), while the Tan, Midnightblue, Blue, and Brown modules were significantly associated with 4 h, 8 h, 12 h, and 16 h of stress, respectively. The Tan module exhibited the highest positive correlation with 4 h stress (r = 0.88, *p* = 1 × 10^−6^) and the weakest negative correlation with 1 h stress (r = −0.53, *p* = 0.02), indicating that its gene set serves as a key regulator of the heat stress response across the six time points (Figure 4a).

A co-expression regulatory network was constructed using 488 genes from the Blue module to identify key regulatory genes across all time points (Figure 4b). Network analysis revealed five pivotal genes—*Hsf8*, *HSP70*, *MBF1C*, *DREB2B*, and *PIF4*—located at critical regulatory nodes. These findings highlight the stage-specific regulatory patterns of *C. lanceolata* under heat stress, such as the significantly positively correlated gene set during the 4-h response phase. Functional characterization of these key regulatory genes will further elucidate the heat adaptation mechanisms in *C. lanceolata*.

### 2.6. Analysis of Multi-Level Hierarchical Regulatory Network Between Transcription Factors and Target Genes

Transcription factors play important roles in *C.lanceolata*’s response to heat stress through co-expression networks. Analyzing their relationships with target genes helps reveal adaptation mechanisms. Under heat stress, families like *bZIP*, *Hsf*, *MYB*, and *SNF2* show different expression patterns. Most transcription factors have low expression levels, while *AP2*, *bHLH*, *Hsf*, and *MBF1* are highly expressed. *AP2*, *Hsf*, and *MBF1* peak at 1 h, and *bHLH* peaks at 8–12 h, indicating temporal specificity (Figure S3).

Based on RNA-seq predicted transcription factor expression levels and target gene binding predictions for major transcription factors [5], a multi-level regulatory network across time points for abiotic stress was constructed using the Bottom-up GGM algorithm. This network clarifies hierarchical regulatory relationships between transcription factors and target genes, providing a theoretical basis for in-depth studies on abiotic stress molecular mechanisms [6]. The high-temperature stress regulatory network reveals two main hubs: one centered on *Hsf8* and *RPT2A* (Figure 4c).

The regulatory network is organized in a three-level architecture: the first and second tiers consist of transcription factors, while the third level comprises target genes. High temperature induces the core transcription factor *Hsf8*, which regulates downstream transcription factors (e.g., *HSP70*, *DREB2B*) to act on target genes such as *RPL24B* and *CALM3*. Meanwhile, heat-induced *RPT2A* regulates transcription factors (e.g., *CAX3*, *PIF4*) to control target genes including *SGT1A* and *HSP70-9*. Genes like *NHX7* and *CAX1* participate in both networks, indicating that *Hsf8* and *RPT2A* coordinately mediate heat adaptation in *C. lanceolata* by co-regulating transcription factors and target genes.

### 2.7. Gene Cloning, Sequence Analysis, and Subcellular Localization Assays

Based on our previous transcriptomic co-expression network analysis, *ClHsf8* was identified as a key transcription factor involved in the heat stress response of *C. lanceolata*. We cloned and functionally characterized this gene. The *ClHsf8* gene contains a 1212 bp coding sequence (CDS) encoding a protein of 403 amino acids. Protein domain prediction indicated that it belongs to the *Hsf* superfamily, with a DNA-binding domain located between amino acids 39 and 131. The predicted molecular weight of *ClHsf8* is 51.04 kDa, with an isoelectric point of 5.64, a GRAVY value of −0.802 indicating that it is a hydrophilic protein, and an aliphatic index of 55.21.

**Figure 5:**
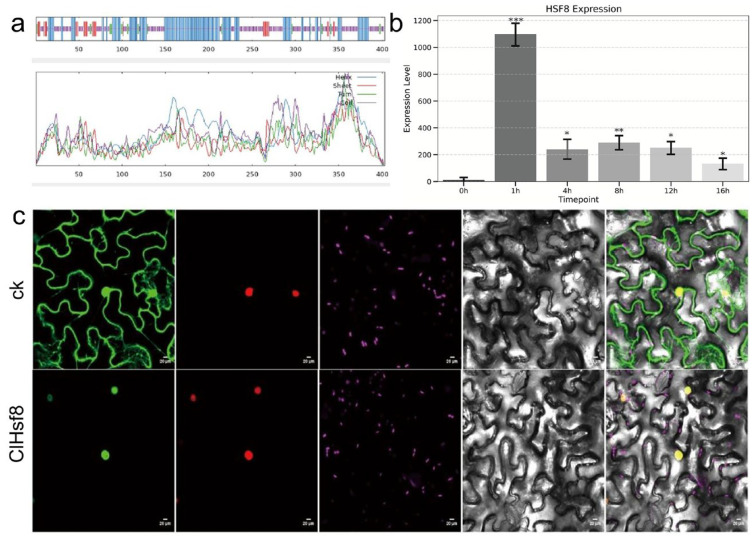
Structural characteristics, expression pattern, and subcellular localization of *ClHsf8*. (**a**) Predicted secondary and tertiary structures of the *ClHsf8* protein. (**b**) Gene expression levels of *ClHsf8* under different conditions. Statistically significant differences were assessed using ANOVA test (* *p* < 0.05, ** *p* < 0.01, *** *p* < 0.001). (**c**) Subcellular localization of ClHsf8. From left to right: fluorescence of the target protein (ClHsf8-GFP fusion) and empty vector control, fluorescence of the nuclear marker (DAPI), chloroplast autofluorescence, bright-field, and merged composite images. Scale bar = 20 μm.

Expression profiling revealed that *ClHsf8* was significantly upregulated under heat stress, particularly during the early stages of treatment, suggesting its potential role as an early-response regulator in the heat stress signaling pathway (Figure 5b). To further investigate its function and subcellular localization, we constructed a 35S:*ClHsf8*-GFP recombinant plasmid and transiently expressed it in tobacco leaves via Agrobacterium-mediated transformation. Confocal laser scanning microscopy revealed that *ClHsf8* is predominantly localized in the nucleus (Figure 5c).

### 2.8. Generation of Transgenic Tobacco and qRT-PCR Analysis Under Heat Stress

To investigate the function of *ClHsf8*, we generated tobacco plants overexpressing this gene via *Agrobacterium*-mediated transformation, yielding multiple transgenic lines. Following kanamycin selection, positive transformants were confirmed through genomic DNA molecular detection. qRT-PCR analysis of 10 randomly selected, vigorously growing independent lines was performed to assess gene expression levels (Figure S4).

**Figure 6:**
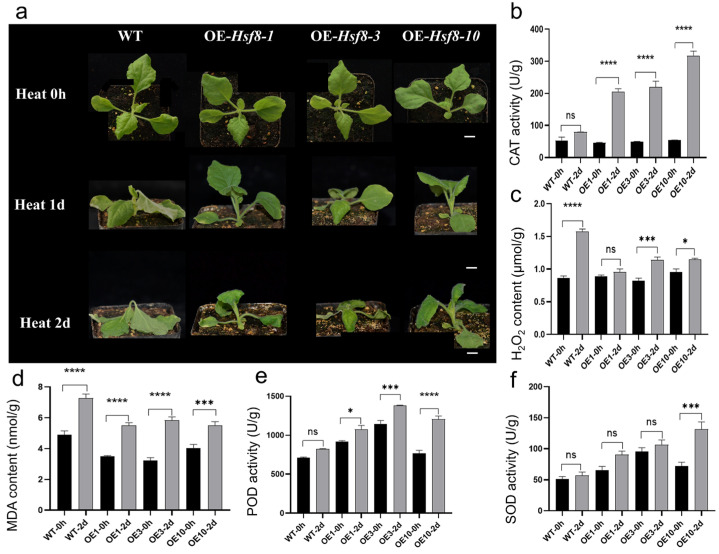
Phenotypic and Physiological Responses of Wild-Type and T1 Transgenic Tobacco under Heat Stress. (**a**) Phenotypes after 2 Days of Heat Treatment; (**b**–**f**) Analysis of Physiological Indices (CAT, H_2_O_2_, MDA, POD, and SOD, respectively). Statistical significance was determined by *t*-test (* *p* < 0.05, *** *p* < 0.001, **** *p* < 0.0001).

To evaluate whether overexpression of *ClHsf8* enhances heat tolerance, three transgenic tobacco lines with high expression levels (*ClHsf8*-1, *ClHsf8*-3, and *ClHsf8*-10) were selected for functional validation. Under heat stress, transgenic plants exhibited distinct phenotypic differences compared to wild-type (WT) plants (Figure 6). After one day of stress, WT plants showed mild leaf edge curling and leaf drooping. In contrast, *ClHsf8*-3 plants displayed only minor drooping near the soil surface, while *ClHsf8*-1 and *ClHsf8*-10 plants remained morphologically unchanged. After two days of stress, WT plants showed severe wilting, pronounced leaf curling, and partial necrosis. *ClHsf8*-3 plants exhibited wilting and necrosis in small basal leaves, whereas *ClHsf8*-1 and *ClHsf8*-10 plants maintained mostly upright leaves, with only slight bending compared to day 1. These observations indicate that *ClHsf8* overexpression significantly enhances heat tolerance in tobacco, with *ClHsf8*-10 exhibiting the strongest resistance among the transgenic lines (Figure 6a).

To further evaluate this phenotype, five physiological parameters—SOD, peroxidase (POD), MDA, catalase (CAT), and Hydrogen peroxide (H_2_O_2_)—were measured in WT and transgenic plants subjected to heat stress. SOD activity was higher in transgenic plants than in WT under both control and heat stress conditions, with *ClHsf8*-10 showing the most pronounced increase after stress. POD activity increased in all plants as heat stress progressed, but the rise was greater in transgenic lines than in WT, with *ClHsf8*-10 again showing the largest increase (Figure 6).

MDA content, which reflects membrane lipid peroxidation, was substantially higher in WT plants than in transgenic lines, indicating greater cellular damage in WT. *ClHsf8*-10 accumulated the least MDA among the transgenic lines, suggesting the lowest level of oxidative damage. CAT activity, another key antioxidant indicator, also increased under stress, with transgenic lines showing a much larger increase than WT. After heat treatment, CAT activity was significantly higher in *ClHsf8* lines, especially *ClHsf8*-10. As an indicator of ROS accumulation, H_2_O_2_ levels also increased under heat stress, but the rise was significantly greater in WT plants compared to transgenic lines, indicating more efficient ROS scavenging in *ClHsf8* overexpressing plants (Figure 6).

Collectively, these results demonstrate that *ClHsf8* overexpression enhances thermotolerance in tobacco by modulating antioxidant enzyme activity and reducing oxidative stress, with *ClHsf8*-10 showing the most robust heat resistance among the tested lines.

## 3. Discussion

### 3.1. Long-Term Heat Stress Can Cause Damage or Even Death to Seedlings

Heat stress significantly affects *C. lanceolata* seedlings, causing wilting, retarded leaf growth, accelerated water loss, leaf curling, and tissue damage. In this study, continuous exposure to 39 °C induced progressive injury. Leaves began to show yellowing after 16 h, followed by severe wilting and desiccation as the stress duration increased. These results demonstrate that prolonged high-temperature stress overwhelms the tolerance and regulatory capacity of Chinese fir.

As in other plants, heat stress disrupts biomolecules, disturbs physiological balance, promotes harmful substance accumulation, and damages cellular structures and defense systems, ultimately leading to seedling mortality. Mechanistically, *C. lanceolata* modulates stomatal aperture to regulate transpiration under heat stress, suggesting an active physiological response to facilitate transpirational cooling. However, our observations revealed that prolonged exposure to 39 °C resulted in the melting of epidermal wax, which subsequently blocked the stomata. This stomatal sealing likely impedes transpiration-mediated cooling, leading to elevated leaf temperature, irreversible cellular damage, and eventual plant death.

### 3.2. Functional Analysis of Differentially Expressed Genes Under High-Temperature Stress

Global warming has increased the frequency of extreme weather events, such as severe drought and high-temperature stress, which pose significant threats to plant survival [7]. Consequently, research on plant thermotolerance has become an increasingly critical area of study. High-temperature stress leads to the deactivation of chlorophyll, reduces photosynthetic rates, and accelerates water loss, resulting in desiccation and ultimately death [8]. For large-scale cultivated tree species such as *C. lanceolata*, extreme heat can be lethal, especially in wild environments where external water supply is unavailable. Therefore, elucidating the adaptive mechanisms of plants under high-temperature stress is essential.

Typically, high-temperature stress signals are perceived by cell membrane receptors, triggering the synthesis of secondary messengers. Heat shock transcription factors (Hsfs), mediated by the heat shock protein (Hsp) cascade, act as central regulators of signal transduction [9], linking changes in secondary messengers to cellular responses. In this study, we constructed 18 libraries and employed RNA-seq to explore gene expression patterns at five time points under high-temperature stress in *C. lanceolata*. We aligned the RNA-seq data to the full-length transcriptome and compared each time point with the control to precisely identify and analyze differentially expressed genes (DEGs). GO enrichment analysis highlighted processes such as cell morphogenesis, unfolded protein binding, phosphotransferase activity, stress response, protein folding, and DNA-dependent transcriptional regulation. KEGG pathway analysis identified enrichment in protein export, endoplasmic reticulum protein processing, and RNA degradation. A total of 1130 genes were identified as DEGs across all time points under heat stress, with functions primarily enriched in signal transduction, protein folding, the plant MAPK signaling pathway, and the NF-kappa B signaling pathway.

### 3.3. Co-Expression Regulatory Network Analysis of Differentially Expressed Genes Under High-Temperature Stress

High-temperature stress is a complex biological process involving the coordinated regulation of numerous genes. Weighted gene co-expression network analysis (WGCNA) is a systems biology approach used to describe patterns of gene associations across different samples. In this study, we constructed a regulatory network comprising 488 differentially expressed genes (DEGs) across all time points under high-temperature stress. Five genes—*Hsf8*, *HSP70*, *MBF1C*, *PIF4*, and *DREB2B*—were identified as key regulatory nodes in the network, along with 42 transcription factors from families such as *AP2*, *C3H*. In the high-temperature regulatory network, *Hsf8* and *RPT2A* emerged as central nodes. Transcription factors such as *DREB2B*, *PIF4*, *MBF1C*, and *HSP70* collectively participated in the response to high-temperature stress. These results indicate that these transcription factors play major regulatory roles in abiotic stress. Under high-temperature stress, transcription factors like *DREB2B*, *Hsf8*, and *MBF1C* were highly expressed at 1 h, while *PIF4* showed high expression at 8 and 12 h. The high expression of these transcription factors at specific time points regulated downstream target genes, thereby enhancing the thermotolerance of *C. lanceolata*.

### 3.4. Overexpression of ClHsf8 and Thermotolerance

The enhanced heat tolerance observed in *ClHsf8* overexpressing tobacco lines underscores the functional significance of *ClHsf8* as a positive regulator of thermotolerance in *C. lanceolata*. The differential phenotypic responses between transgenic and wild-type plants, particularly under prolonged high-temperature stress, suggest that *ClHsf8* contributes to the maintenance of cellular homeostasis under thermal stress. Notably, the superior performance of ClHsf8-10 indicates that expression levels or positional effects may further modulate the strength of the protective response. The observed increases in antioxidant enzyme activities (SOD, POD, and CAT) and the concomitant reduction in oxidative damage (as evidenced by lower MDA (malondialdehyde) and H_2_O_2_ levels) point toward a *ClHsf8*-mediated enhancement of the antioxidant defense system. This is consistent with previous findings in other plant species, such as *Arabidopsis* where *Hsf* family members activate ROS-scavenging pathways to alleviate heat-induced oxidative stress [10].

Moreover, the early and sustained elevation of antioxidant activities in *ClHsf8* overexpressing lines suggests that *ClHsf8* may act upstream of key ROS detoxification pathways, possibly through direct or indirect regulation of stress-responsive genes. These findings align with the classical role of *Hsfs* as transcriptional activators that bind to heat shock elements (HSEs) and initiate transcription of target genes involved in heat adaptation. It is therefore plausible that *ClHsf8* regulates a subset of heat-inducible genes, including ROS-scavenging enzymes, chaperones, or signaling components, thereby enhancing cellular resilience. Further investigations, such as ChIP-seq or transcriptome profiling of *ClHsf8*-overexpressing lines, could help delineate the direct targets and broader regulatory network of *ClHsf8*.

In summary, our findings provide strong evidence that *ClHsf8* functions as a key transcriptional regulator in the heat stress response, conferring enhanced thermotolerance by strengthening the antioxidant defense machinery. These results not only expand our understanding of heat stress regulatory mechanisms in conifers but also offer a potential genetic resource for engineering stress-resilient forestry species.

## 4. Materials and Methods

### 4.1. Sample Collection and High-Temperature Stress Treatment

The elite clone ‘6421’ of *C. lanceolata*, widely cultivated in southern China for its fast growth and high timber yield, was selected as the experimental material. Seedlings were obtained from Yangkou State-owned Forest Farm (Nanping, China). The plant materials consisted of 3-month-old seedlings grown in plastic pots filled with a mixture of vermiculite and nutrient soil (1:2 *v*/*v*). Plants were watered every 3 days to maintain adequate moisture and prevent drought stress prior to the high-temperature treatment. Before the stress experiments, seedlings were acclimated in a high-precision plant growth chamber under a 25 °C/22 °C (day/night) cycle, with 75% relative humidity, 5500 lx light intensity, and a 16 h light/8 h dark photoperiod.

To apply the high-temperature stress, the growth chambers were pre-heated to the target temperatures. Seedlings were subjected to two distinct heat stress regimes: 39 °C (representing sub-lethal stress for transcriptomic analysis) and 42 °C (representing lethal stress for phenotypic contrast). To ensure the validity of the constant stress treatment, the internal temperature was continuously monitored using independent sensors and maintained strictly at 39 °C ± 0.5 °C (or 42 °C ± 0.5 °C) throughout the experiment to eliminate environmental fluctuations. For the transcriptomic study (39 °C), a reverse time course design was employed to avoid circadian rhythm effects. Plants assigned to different stress durations (1, 4, 8, 12, and 16 h) were transferred into the chamber at staggered intervals, allowing all samples, including the control (0 h), to be harvested simultaneously. For phenotypic observation and physiological measurements, the stress duration was extended to 24 h, 2 d, and 3 d to capture the cumulative damage and long-term heat injury progression.

After high-temperature treatment, the phenotypic response of *C. lanceolata* seedlings was quantitatively evaluated using an observation-based scoring method. Healthy seedlings were selected before treatment, and the total number of leaves per plant was recorded. After each stress duration, individual leaves were assigned a heat damage score based on visible symptoms: leaf tip yellowing = 0.6, 10–40% yellowing area = 0.8, and >40% yellowing = 1. A heat injury index was then calculated to quantify the overall degree of leaf damage. For cellular ultrastructure analysis, functional leaves from the middle canopy were sampled. Leaf tissues were sectioned and fixed in 4% glutaraldehyde, vacuum-infiltrated at 4 °C for 24 h, then washed with PBS and dehydrated in an ethanol series. Samples for scanning electron microscopy (SEM) were gold-coated and imaged using a Hitachi SEM (Tokyo, Japan). For transmission electron microscopy (TEM), samples were post-fixed in osmium tetroxide, embedded in resin, sectioned, and double-stained with uranyl acetate and lead citrate. Imaging was performed using a Hitachi HT7700 TEM (Tokyo, Japan).

### 4.2. Phenotypic Observation and Assessment of Heat Injury Index

To quantify the phenotypic changes after heat stress, the Heat Injury Index was calculated using an observation and counting method. For each selected healthy seedling, the total number of leaves was initially recorded as *Z*. Following each heat treatment duration, every leaf was assigned a specific heat injury score based on the severity of yellowing: a score of 0 was given to healthy leaves with no visible yellowing; 0.6 for leaves exhibiting only tip yellowing; 0.8 for leaves with a yellowing proportion between 10% and 40% of the total leaf area; and 1.0 for leaves with a yellowing proportion greater than 40%. Finally, the overall Heat Injury Index for each seedling was calculated using the formula:

$$Heat\;Injury\;Index\left(\%\right)=\frac{\left(\sum Heat\;injury\;score\;of\;each\;leaf\right)}{Z}\times 100$$

### 4.3. Determination of Physiological Indices Under Heat Stress

To investigate the physiological responses of *C. lanceolata* tissue culture seedlings under heat stress, several physiological parameters were measured, including chlorophyll content, MDA, proline, SOD, and POD activities. Leaf samples were immediately frozen in liquid nitrogen after collection and stored at −80 °C until analysis.

Chlorophy II content was determined using ethanol extraction followed by spectrophotometric analysis. MDA content was measured using the thiobarbituric acid (TBA) method, while proline was quantified via the ninhydrin colorimetric assay. SOD and POD activities were assessed using Jiancheng commercial assay kits (Nanjing, China). Three biological replicates were included for each treatment time point.

### 4.4. RNA Extraction and Transcriptome Sequencing and Transcriptome Data Analysis

Total RNA was extracted using the Vazyme RNA kit (Nanjing, China). RNA quality was checked by 1% agarose gel electrophoresis and an Agilent 2100 Bioanalyzer. Poly(A) + mRNA was enriched using Oligo (dT) beads and reverse transcribed into cDNA. After end repair and adapter ligation, sequencing libraries were constructed and sequenced on the Illumina NovaSeq 6000 platform with 150 bp paired-end reads. Clean reads were aligned to the full-length transcriptome using Kallisto [11] (v0.46.1) to calculate TPM values. Differentially expressed genes (DEGs) were identified with DESeq2 [12] (v1.16.1) in R (v4.0.3) and visualized using ggplot2 (v3.3.6). GO and KEGG enrichment analyses of DEGs were performed with ClusterProfiler [13] (v3.18.0). Weighted gene co-expression network analysis [14], v1.69 was applied to construct co-expression networks after outlier removal, with a soft threshold of 9 (correlation > 0.8) and a maximum module number of 5000. Modules showing the most significant differences between control and treatment time points were selected and visualized with Cytoscape (v3.7.2). Gene expression heatmaps were generated using Pheatmap (v1.0.12).

### 4.5. Construction of Multilayer Hierarchical Gene Regulatory Network Under Heat Stress

A multilayer hierarchical gene regulatory network (Ml-hGRNs) was constructed using the Bottom-up GGM algorithm [15] based on RNA-seq time-series expression data to analyze regulatory relationships between transcription factors (TFs) and target genes. Stress-related TFs and their corresponding target gene expression data were first selected, followed by multilayer network construction using the algorithm. The regulatory network was visualized using the online platform (http://sys.bio.mtu.edu/topdown/bottomUp.php (accessed on 15 May 2025)).

### 4.6. Molecular Cloning, Expression Profiling, and Structural Prediction of ClHsf8

The full-length cDNA sequence of *ClHsf8* was amplified by PCR using gene-specific primers designed based on the transcriptome data (see Table S1 for primer sequences). PCR reactions were prepared on ice, containing the cDNA template, reaction mix, buffer, specific primers, and DNA polymerase. The amplification protocol consisted of an initial denaturation at 95 °C for 3 min, followed by 35 cycles of 95 °C for 15 s, annealing at the primer-specific temperature for 15 s, and extension at 72 °C for 30–60 s/kb, with a final extension at 72 °C for 5 min.

For gene expression analysis under heat stress, *C. lanceolata* tissue culture seedlings were subjected to 42 °C treatment, and young leaves from the upper part of the seedlings were collected at 1 h, 4 h, 8 h, 12 h, and 16 h. Untreated seedlings served as the control group. Total RNA was extracted and reverse-transcribed into cDNA. Quantitative real-time PCR (qRT-PCR) was performed using a Roche LightCycler^®^ 480II system and 2 × AceQ^®^ qPCR SYBR^®^ Green Master Mix (Nanjing, China). The *C. lanceolata eIF-3* gene was used as the internal reference for normalization. The cycling conditions were: 95 °C for 30 s, followed by 45 cycles of 95 °C for 10 s and 60 °C for 30 s. Each sample was analyzed with three biological replicates and three technical replicates. Relative expression levels were calculated using the $2^{-\Delta \Delta CT}$ method.

Additionally, conserved domain analysis was performed using the NCBI Conserved Domain Database (CDD) (accessed on 15 May 2025). Protein physicochemical properties were predicted using ExPASy ProtParam (accessed on 15 May 2025), and secondary structures were predicted using SOPMA (accessed on 15 May 2025).

### 4.7. Subcellular Localization Analysis

The GFP fusion vector for *ClHsf8* was constructed using homologous recombination combined with Golden Gate seamless cloning, employing BsaI and Eco3II restriction enzymes to generate compatible cohesive ends for seamless ligation. Primers were designed using SnapGene (v4.18) (Table S2). The recombinant plasmid pBWA(V)HS-*ClHsf8* -Glosgfp was assembled via homologous recombination and subsequently transformed into Agrobacterium tumefaciens strain EHA105 for further use.

Tobacco seeds were grown for one month under a 12 h light cycle. Recombinant plasmids were introduced into EHA105 by electroporation and cultured at 30 °C for 2 d. The bacteria were then transferred to 10 mL YEB medium and shaken at 170 rpm for 1 h. Cells were collected by centrifugation, resuspended in 10 mM MgCl_2_ with 120 µM acetosyringone (AS), and adjusted to an OD_600_ of 0.6. Healthy tobacco plants were selected, and the bacterial suspension was injected into the lower leaf epidermis using a needleless syringe with proper labeling. Plants were incubated under low light for 2 d. Marked leaves were prepared as slides and observed by confocal microscopy with image capture. For colocalization, marker and recombinant plasmids were cotransformed into Agrobacterium, and suspensions were mixed 1:1 before injection.

### 4.8. Overexpression Vector pRI101 Construction

The overexpression vector pRI101 was constructed by homologous recombination. The pRI101 vector was linearized using Sal I and Xma I restriction enzymes at 37 °C for 2 h, followed by heat inactivation at 65 °C for 1 h. Primers, including a 35S forward primer and a gene-specific reverse primer, were designed with SnapGene (Table S3). PCR amplification was performed using Vazyme Phanta High-Fidelity DNA Polymerase (Nanjing, China). The digested vector and insert fragment were ligated at 37 °C for 30 min to generate the recombinant overexpression vector.

### 4.9. Agrobacterium-Mediated Genetic Transformation of Tobacco

Tobacco seeds were sterilized with 75% ethanol and 84 disinfectant, rinsed with sterile water, and germinated on MS medium under a 16 h light/8 h dark cycle at 23 °C for 4–5 weeks. Sterile leaves were excised and pre-cultured before being inoculated with Agrobacterium (OD_600_ = 0.2) for 10–15 min. After drying, leaves were co-cultivated in the dark for 48–72 h, then transferred to induction medium for callus formation (~10 days). Calli were selected on antibiotic-containing medium for 15–30 d, then moved to differentiation medium under the same light conditions. Regenerated shoots were transferred to nursery medium for 7–10 d. Positive transformants were identified by PCR using genomic DNA extracted with the CTAB method. Verified plants were acclimatized in a peat moss: perlite substrate, covered with plastic wrap for two weeks to maintain humidity before transfer to normal growth conditions.

### 4.10. Measurement of Physiological Parameters Under High-Temperature Stress

To assess the role of *ClHsf8* in heat tolerance, 2-week-old T1 transgenic tobacco plants and wild-type (WT) controls were exposed to heat stress at 42 °C under a 16 h light/8 h dark cycle with 75% relative humidity. After 24 h treatment, leaf samples were collected, flash-frozen in liquid nitrogen, and stored at −80 °C. The activities of SOD, POD, and CAT, as well as the contents of MDA and H_2_O_2_, were quantified using Jiancheng commercial assay kits (Nanjing, China). All measurements were performed with three biological replicates.

## 5. Conclusions

Under high-temperature conditions, *C. lanceolata* activates a multilayered defense mechanism in which *ClHsf8* plays a central regulatory role. As a heat-responsive transcription factor, *ClHsf8* orchestrates the expression of heat shock proteins and modulates antioxidant pathways. Overexpression of *ClHsf8* enhances the activities of key antioxidant enzymes such as SOD and POD, improves reactive oxygen species (ROS) scavenging, and reduces MDA accumulation, thereby alleviating membrane lipid peroxidation. These physiological adjustments contribute to improved cellular stability, reduced leaf wilting, and better maintenance of photosynthetic integrity under heat stress. This study provides a comprehensive mechanistic insight into the heat tolerance of the ‘6421’ genotype, highlighting *ClHsf8*-mediated transcriptional regulation and antioxidant defense as pivotal components of thermotolerance. These findings offer valuable genetic targets for breeding heat-resilient coniferous tree species, supporting adaptive forestry in the context of global climate change.

## Data Availability

The heat-stress transcriptome (RNA seq) dataset for *C. lanceolata* has been deposited in the National Genomics Data Center (NGDC) under accession number CRA034582. All other supplementary data are available in the Supplementary Figures and Tables File. The *C. lanceolata* plant material has been authorized for use by the Yangkou State-owned Forest Farm of Fujian Province.

## Supplementary Materials

The following supporting information can be downloaded at: https://www.mdpi.com/article/10.3390/plants15081150/s1, Figure S1: Physiological Responses of C. lanceolata Leaves to Different Heat Stress Treatments. (a–d) represent chlorophyll content, malondialdehyde (MDA) content, superoxide dismutase (SOD) activity, and proline content, respectively. Statistical significance was determined by T-test: * *p* < 0.05, ** *p* < 0.01, *** *p* < 0.001; Figure S2: Correlation analysis (a) and Principal component analysis (b) of high temperature stress samples; Figure S3: Expression pattern of main Clion factors under high temperature stress; Figure S4: Gel Electrophoresis of genomic PCR results of transgenic plants; Table S1: Primer sequences for gene cloning; Table S2: Primer sequences for vector construction; Table S3: Primer sequences used in gene recombination vector; Table S4: List of RNAsequencing data output quality.
