# Supplementary material for: Transcriptomic and Functional Characterization of ClHsf8 Reveals Key Mechanisms of Heat Stress Response in Cunninghamia lanceolata
Source: Plants (Basel). 2026 Apr 9;15(8):1150. doi: 10.3390/plants15081150 (PMC13119729; doi:10.3390/plants15081150)
Supplement: Supplementary file 1 [file plants-15-01150-s001.zip › plants-4196911-supplementary.pdf]

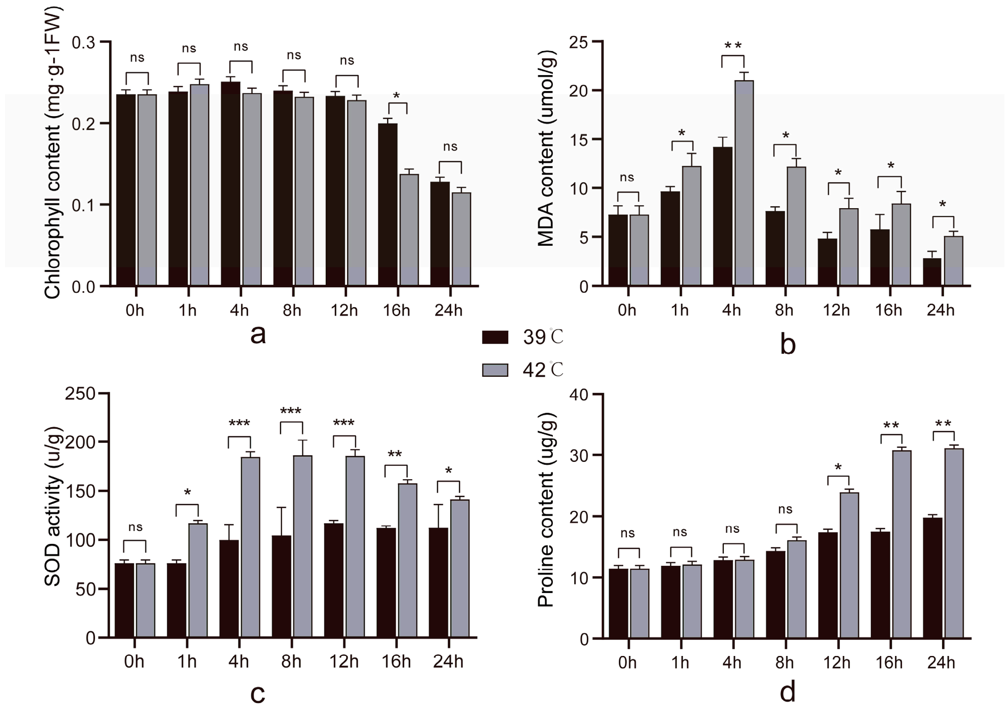

Figure S1 Physiological Responses of *C. lanceolata* Leaves to Different Heat Stress Treatments. (a)-(d) represent chlorophyll content, malondialdehyde (MDA) content, superoxide dismutase (SOD) activity, and proline content, respectively. Statistical significance was determined by T-test:$*P<0.05$, $**P < 0.01$, $***P < 0.001$.

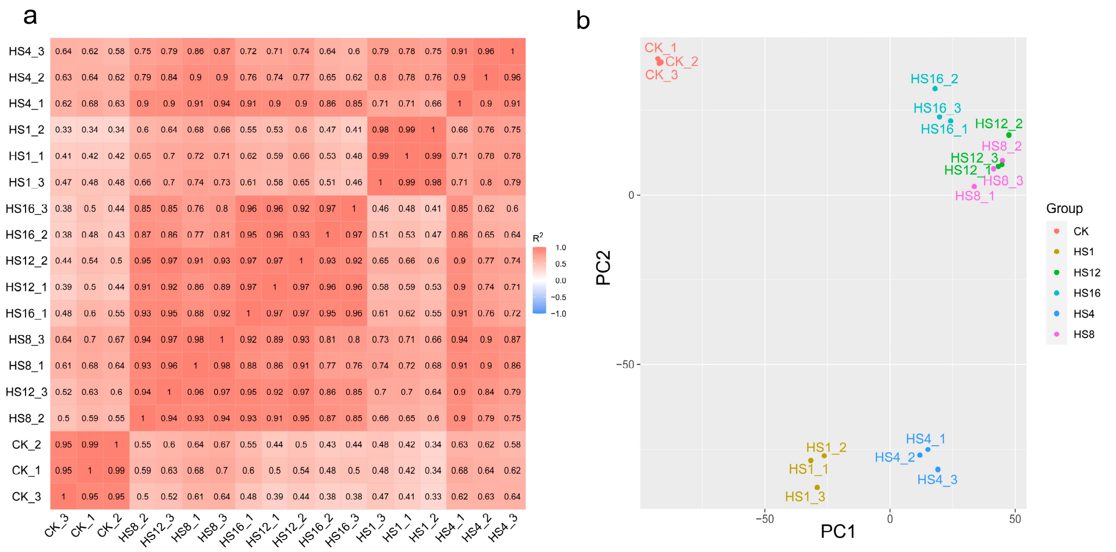

Figure S2 Correlation analysis **(a)** and Principal component analysis **(b)** of high temperature stress samples

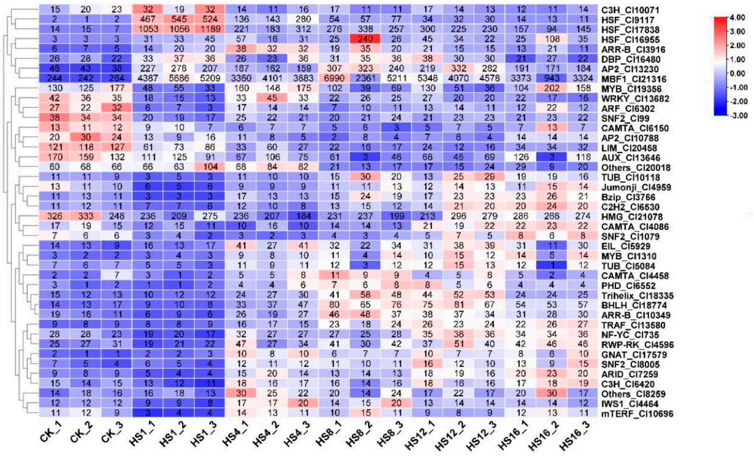

Figure S3 Expression pattern of main Clion factors under high temperature stress

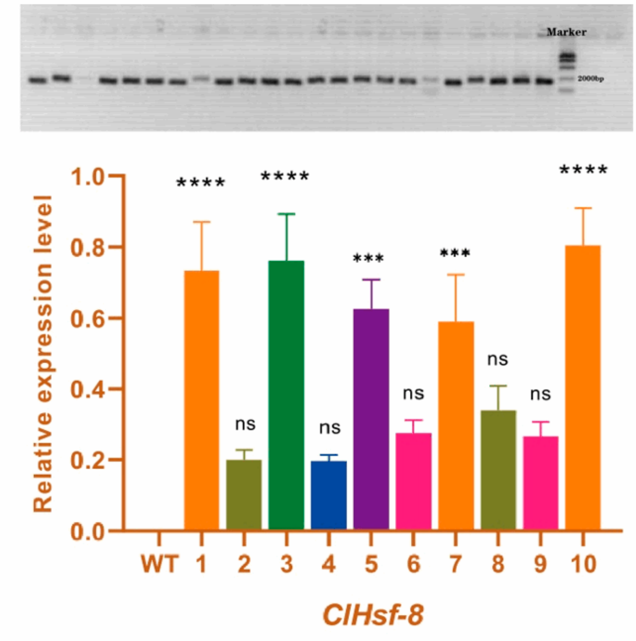

Figure S4 Gel Electrophoresis of genomic PCR results of transgenic plants

Table S1 Primer sequences for gene cloning

Primer	sequence（5′-3′）
ClHSF-08F	ATGGATTCGGGCTTTTGTCAGG
ClHSF-08R	TTAGGGTGGGAGAAAATTGGGTTC
ClHSP70-14F	ATGGCGAAGGGCGGAGGAAAGGCCGTGG
ClHSP70-14R	TTAATCGACTTCCTCAATTTTGGGTCCA

Table S2 Primer sequences for vector construction

Primer	Sequence（5′-3′）
ClHSF-8F	cagtGGTCTCacaacatggattcgggcttttgtcagggg
ClHSF-8R	cagtGGTCTCatacagggtgggagaaaattgggttccac
ClHSP70-14F	cagtGGTCTCacaacatggcgaagggcggaggaaag
ClHSP70-14R	cagtGGTCTCatacaatcgacttcctcaattttgggtccagc

Table S3 Primer sequences used in gene recombination vector

Primer	Sequence（5′-3′）
pRI-ClHSF-08-F	tgttgatacatatgcccgttATGGATTCGGGCTTTTGTCAGG
pRI-ClHSF-08-R	tcggatccggtacccTTAGGGTGGGAGAAAATTGGGTTCC
pRI-ClHSP70-14 F	tcttcactgttgatacatatgcccgttATGGCGAAGGGCGGAG
pRI-ClHSP70-14 R	ATCGATgaattcggatccggtacccttATCGACTTCCTCAATTTTGGGTCC

Table S4 List of RNAsequencing data output quality

Sample	Raw Reads	Clean Reads	Raw Base (G)	Clean Base (G)	Effective Rate (%)	Q20 (%)	Q30 (%)	GC Content (%)
H_12h_1	21,359,127	20,317,527	6.4	6.1	95.12	98.25	94.38	43.11
H_8h_2	28,588,189	27,416,654	8.5	8.2	95.90	97.64	93.09	42.98
H_16h_2	22,186,015	20,857,511	6.6	6.2	94.01	98.03	93.85	42.12
H_0h_1	26,735,731	25,682,785	8.0	7.7	96.06	97.75	93.29	43.59
H_1h_2	26,555,911	24,630,453	7.9	7.3	92.75	97.78	93.46	43.85
H_1h_3	24,660,411	22,668,111	7.4	6.8	91.92	97.78	93.48	44.24
H_0h_3	25,427,217	23,679,691	7.6	7.1	93.13	97.82	93.53	43.66
H_16h_3	32,911,257	30,402,173	9.8	9.12	92.38	97.72	93.31	42.45
H_4h_1	27,676,801	25,554,084	8.3	7.6	92.33	97.78	93.42	42.98
H_4h_2	26,461,717	25,376,544	7.9	7.6	95.90	97.72	93.26	43.61

H_16h_1	23,327,742	21,741,171	7.0	6.5	93.20	97.79	93.34	42.58
H_8h_3	26,523,243	25,248,747	7.9	7.5	95.19	97.80	93.46	43.05
H_12h_2	23,980,625	22,343,760	7.1	6.7	93.17	98.25	94.40	42.16
H_12h_3	24,493,391	22,994,597	7.3	6.9	93.88	97.87	93.50	42.47
H_0h_2	25,532,005	24,084,700	7.6	7.2	94.33	97.79	93.42	43.30
H_1h_1	29,272,941	27,500,195	8.7	8.2	93.94	97.75	93.41	43.73
H_4h_3	30,235,940	28,902,602	9.0	8.6	95.59	97.68	93.18	43.26
H_8h_1	28,658,339	27,573,326	8.6	8.2	96.21	97.58	92.94	43.47
